# Inhibitory Effects of Trapping Agents of Sulfur Drug Reactive Intermediates against Major Human Cytochrome P450 Isoforms

**DOI:** 10.3390/ijms18071553

**Published:** 2017-07-20

**Authors:** Jasleen K. Sodhi, Erlie Marie Delarosa, Jason S. Halladay, James P. Driscoll, Teresa Mulder, Patrick M. Dansette, S. Cyrus Khojasteh

**Affiliations:** 1 Department of Drug Metabolism and Pharmacokinetics, Genentech, Inc., 1 DNA Way (MS 412a), South San Francisco, CA 94080, USA; erlie.delarosa@gmail.com (E.M.D.); jhalladay@plexxikon.com (J.S.H.); jdriscoll@myokardia.com (J.P.D.); dong.teresa@gene.com (T.M.); khojasteh.cyrus@gene.com (S.C.K.); 2 Department of Bioengineering and Therapeutic Sciences, University of California San Francisco, San Francisco, CA 94143, USA; 3 Department of Drug Metabolism and Pharmacokinetics, MicroConstants, Inc., San Diego, CA 92121, USA; 4 Department of Drug Metabolism and Pharmacokinetics, Plexxikon Inc., Berkeley, CA 94710, USA; 5 Department of Drug Metabolism and Pharmacokinetics, MyoKardia, Inc., South San Francisco, CA 94080, USA; 6 Laboratoire de Chimie et Biochimie Pharmacologiques et Toxicologiques, UMR 8601 CNRS, Université Paris Descartes, Sorbonne Paris Cité, 75270 Paris CEDEX 06, France; patrick.dansette@parisdescartes.fr

**Keywords:** cytochrome P450, bioactivation, trapping agents, reactive metabolites, CYP inhibition, sulfur drug reactive intermediates

## Abstract

In some cases, the formation of reactive species from the metabolism of xenobiotics has been linked to toxicity and therefore it is imperative to detect potential bioactivation for candidate drugs during drug discovery. Reactive species can covalently bind to trapping agents in in vitro incubations of compound with human liver microsomes (HLM) fortified with β-nicotinamide adenine dinucleotide phosphate (NADPH), resulting in a stable conjugate of trapping agent and reactive species, thereby facilitating analytical detection and providing evidence of short-lived reactive metabolites. Since reactive metabolites are typically generated by cytochrome P450 (CYP) oxidation, it is important to ensure high concentrations of trapping agents are not inhibiting the activities of CYP isoforms. Here we assessed the inhibitory properties of fourteen trapping agents against the major human CYP isoforms (CYP1A2, 2C9, 2C19, 2D6 and 3A). Based on our findings, eleven trapping agents displayed inhibition, three of which had IC_50_ values less than 1 mM (2-mercaptoethanol, *N*-methylmaleimide and *N*-ethylmaleimide (NEM)). Three trapping agents (dimedone, *N*-acetyl-lysine and arsenite) did not inhibit CYP isoforms at concentrations tested. To illustrate effects of CYP inhibition by trapping agents on reactive intermediate trapping, an example drug (ticlopidine) and trapping agent (NEM) were chosen for further studies. For the same amount of ticlopidine (1 μM), increasing concentrations of the trapping agent NEM (0.007–40 mM) resulted in a bell-shaped response curve of NEM-trapped ticlopidine *S*-oxide (TSO-NEM), due to CYP inhibition by NEM. Thus, trapping studies should be designed to include several concentrations of trapping agent to ensure optimal trapping of reactive metabolites.

## 1. Introduction

The relationship between drug bioactivation in vivo and subsequent toxicity is not straightforward; however, there have been reported instances of drug toxicity directly resulting from reactive metabolite formation [1,2]. For this reason, a major effort during drug discovery is to assess the possibility of reactive metabolite formation in the attempt to minimize the potential of compounds to cause toxicity in humans [3,4]. In vitro trapping studies are routinely performed as an integral part of drug discovery, as they can provide insight into the bioactivation potential of candidate drugs [5,6]. Reactive metabolite formation can be assessed by trapping the unstable reactive intermediate with trapping agents that form stable conjugates with the reactive species [7]. Since hepatic cytochrome P450 (CYP) enzymes are mainly responsible for known bioactivation reactions [8] these assays are typically performed with human liver microsomes (HLM) fortified with reduced β-nicotinamide adenine dinucleotide phosphate (NADPH) [9]. A trapping agent can be co-incubated with the compound of interest in microsomal incubations, thus allowing it to react directly and form stable conjugates with any CYP-mediated reactive metabolites generated. These stable conjugates can then be detected by liquid chromatography-tandem mass spectrometry (LC-MS/MS) [5,10]. Recognizing the role of trapping agents in trapping reactive intermediates in close proximity to the CYP active site, it is possible that trapping agents also inhibit CYP activity, potentially modulating the CYP-mediated metabolism of the compound of interest and underestimating the formation of potential reactive metabolites.

Despite widespread use of trapping agents, their ability to inhibit the major CYP isoforms has not been thoroughly characterized. The selection of trapping agents tested here was based on former studies on sulfur-containing drugs, metabolism of which may lead to reactive sulfenic acid species or thiophene *S*-oxide intermediates (in the case of thiophenes). Both sulfenic acid and thiophene *S*-oxide have been known to covalently modify proteins critical in cell function, potentially resulting in toxicity [11]. Detection of sulfenic acid is especially challenging, as it is a highly elusive and unstable intermediate with a very short half-life (less than 1 min) [12]. It is quite reactive, and can undergo a multitude of reactions resulting in a wide variety of chemical species. For example, sulfenic acid can undergo further oxidations to more stable metabolites (such as sulfinic and sulfonic acids), react with another sulfenic acid (disproportionation reaction) to form a free thiol and a sulfinic acid, react with nucleophiles (like thiols) resulting in mixed disulfides [13], react with cyanide (resulting in thiocyanate), react with thiocyanate (resulting in dithiocyanate) [14], or even react with nucleophilic moieties of proteins, commonly with cysteine residues, to form covalent adducts [11]. Due to this highly reactive behavior of sulfenic acid intermediates, detection is especially challenging.

Despite difficulties in the detection of sulfenic acid and its derivatives directly by LC-MS/MS, the formation of these reactive species can be inferred by adding a trapping agent to an incubation of compound with metabolizing enzymes, which can covalently bind to the metabolically-generated reactive species, resulting in a stable adduct for detection [11]. In 1974, 5,5-dimethyl-1,3-cyclohexanedione (dimedone) was the first agent shown to selectively react with sulfenic acid, resulting in a stable conjugate which facilitated detection of the short-lived sulfenic acid species [15].

Trapping agents are selected to target a specific type of reactive metabolite or reaction and may be nucleophiles, reducing agents, dienophiles, Michaël acceptors, or radical scavengers. For example, sulfenic acid can be trapped by nucleophilic-diketo derivatives such as dimedone and methyl-cyclohexane 1,3-dione (MCD) [16,17]. It can itself act as a nucleophile and react with activated vinyl such as acrylamide and maleimides (Michaël acceptors) [18], or even be reduced to a thiol by ascorbic acid (a radical scavenger) or by the reducing agents arsenite or tris(2-carboxyethyl) phosphine (TCEP) [19,20]. Thiophene derivatives are often metabolized to thiophene *S*-oxides, which can be trapped by thiol nucleophiles (glutathione (GSH), *N*-acetyl-cysteine or mercaptoethanol) or may form Diels Alder dimers [21,22]. Additionally, it has been shown that dienophiles (e.g., maleimides) can be used to trap thiophene *S*-oxide during microsomal incubations [23]. For trapping of other species of reactive metabolites, the agent GSH is commonly used to assess the formation of soft electrophiles, and methoxyamine, semicarbazide and cyanide are used to assess the formation of hard electrophiles such as aldehydes and iminium ions [24]. Therefore, a variety of trapping agents exist which may be used to trap a variety of reactive metabolites and the stability of the conjugates formed between trapping agents and reactive thiol metabolites allows for easier detection than the thiols alone using conventional LC-MS/MS methods.

**Figure 1:**
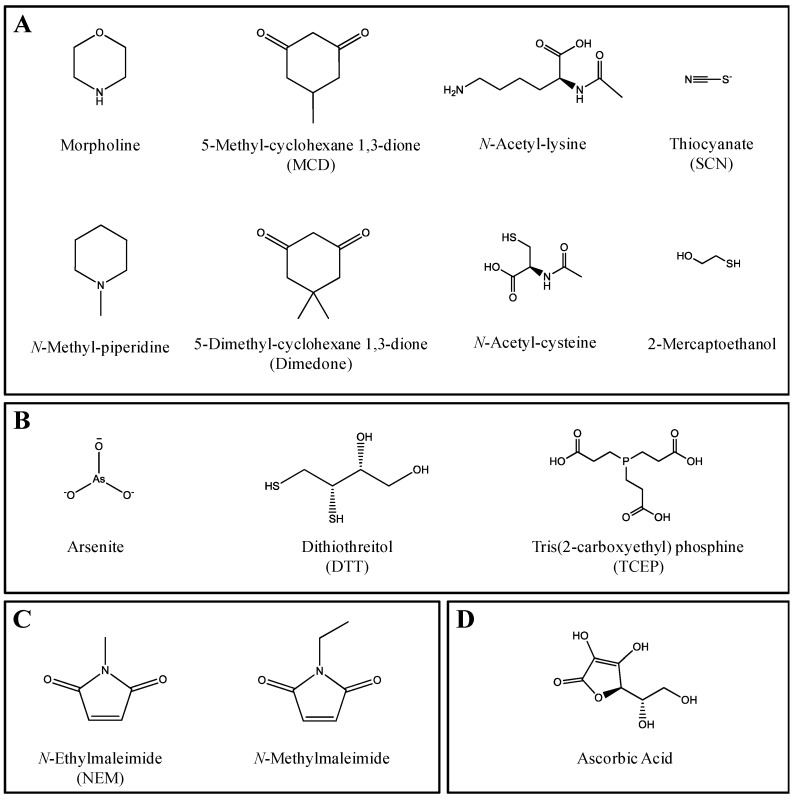
Chemical structures of (**A**) nucleophiles; (**B**) reducing agents; (**C**) Michaël acceptors and dienophiles and (**D**) a radical scavenger.

**Figure 2:**
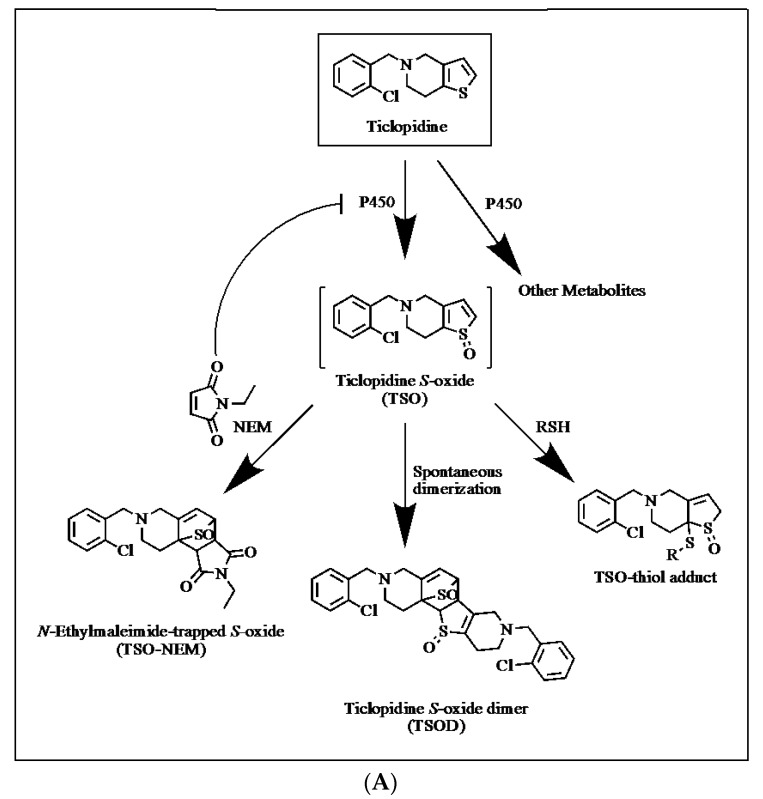

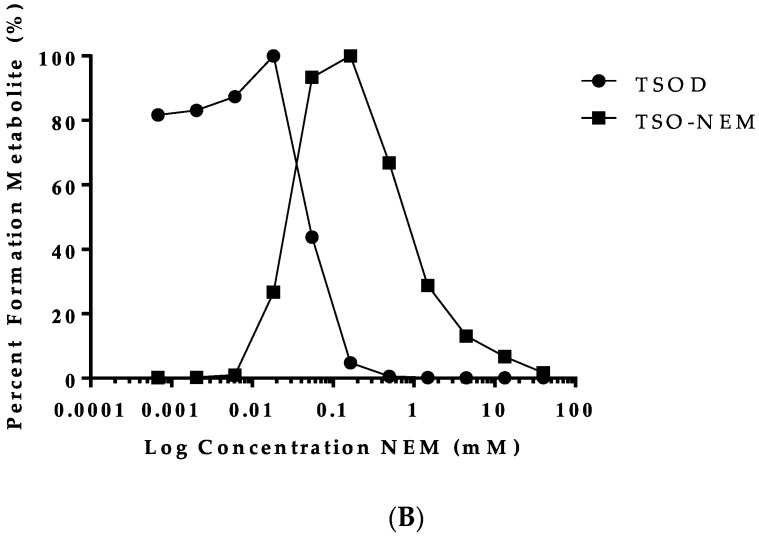
Ticlopidine metabolism in human liver microsomes in the presence and absence of *N*-ethylmaleimide (NEM). (**A**) Proposed metabolic pathways of ticlopidine in human liver microsomes in the presence and absence of *N*-ethylmaleimide (NEM) (**B**) Formation of Ticlopidine *S*-oxide dimer (TSOD) and *N*-ethylmaleimide ticlopidine *S*-oxide adduct (TSO-NEM) following 15 min incubations with ticlopidine (1 µM), human liver microsomes (0.5 mg/mL) fortified with reduced β-Nicotinamide adenine dinucleotide phosphate (NADPH) and various concentrations of NEM (0–40 mM). The reported relative percentages of formation of the metabolites are normalized to the incubation which formed the highest amount (TSOD or TSO-NEM).

The work presented here has the following objectives: (1) to identify the inhibitory potential of fourteen agents (including nucleophiles, reducing agents, Michaël acceptors/dienophiles, and radical scavengers) (Figure 1) commonly used to trap reactive metabolites formed via CYP-mediated metabolism and (2) to demonstrate the effects of CYP inhibition by a trapping agent on the formation and detection of sulfenic acid intermediates, specifically, of a known pathway of ticlopidine metabolism (Figure 2a). Metabolism of ticlopidine results in formation of the reactive ticlopidine *S*-oxide (TSO) species, which quickly dimerizes through a Diels-Alder cycloaddition into a ticlopidine *S*-oxide dimer (TSOD) [25,26]. In this particular investigation, increasing concentrations of a dienophile trapping agent *N*-ethylmaleimide (NEM) were included in an incubation containing ticlopidine and HLM fortified with NADPH. Inclusion of the trapping agent NEM results in a Diels Alder adduct of TSO and NEM (TSO-NEM), preventing TSOD formation [23]. Various concentrations of NEM were tested to examine how the extent of CYP inhibition by the trapping agent NEM affects the amount of trapped and detected reactive ticlopidine metabolite (TSO-NEM). These studies highlight how the challenging task of accurately detecting sulfenic acid and its derivatives due to its high reactivity and short half-life can be further exacerbated by potential CYP inhibition caused by the trapping agent itself. Consideration of the results presented here is integral when designing in vitro trapping studies.

## 2. Results

### 2.1. CYP Inhibition

The IC_50_ data of fourteen trapping agents against major CYP isoforms are outlined in Table 1. Of the eight nucleophiles tested (morpholine, *N*-methylpiperidine, MCD, dimedone, thiocyanate (SCN), 2-mercaptoethanol, *N*-acetyl-cysteine and *N*-acetyl-lysine), all agents except dimedone and *N*-acetyl-lysine showed inhibition against various CYP isoforms at the concentrations tested. Morpholine (typically used in in vitro trapping studies at 10 mM) inhibited the major CYP isoforms (with the exception of CYP1A2) with IC_50_ values ranging from 7.9–16 mM. Although CYP1A2 metabolism was not inhibited by morpholine, interestingly activation was observed. *N*-Methylpiperidine (typically used at 10 mM in in vitro trapping studies) showed inhibition against all CYP isoforms tested, with IC_50_ values (in order of potency) of 6.5 mM (CYP2D6), 9.9 mM (CYP2C9), 12 mM (CYP3A, midazolam probe), 14 mM (CYP3A, testosterone probe), 19 mM (CYP2C19) and ~50 mM (CYP1A2). CYP1A2 metabolism was also activated by *N*-methylpiperidine at concentrations up to 12.5 mM, followed by inhibition up to 100 mM, resulting in an approximate IC_50_ of 50 mM. MCD (typically used in in vitro trapping studies at 4 mM) inhibited all isoforms with the exception of CYP2D6, with IC_50_ values ranging from 3.1–6.7 mM. SCN inhibited major CYP isoforms, with the exception of CYP1A2, at concentrations higher than what is typically used for in vitro trapping studies (4 mM), with IC_50_ values ranging from 18–49 mM. 2-Mercaptoethanol (typically used in vitro at 20 mM) showed inhibition across all CYP isoforms tested, with IC_50_ values (in order of potency) of 0.33 mM (CYP2C19), 2.0 mM (CYP2C9), 7.8 mM (CYP3A; testosterone probe), 8.9 mM (CYP3A; midazolam probe), 24 mM (CYP2D6), and 42 mM (CYP1A2). These inhibition data are noteworthy, as there are reports of using 2-mercaptoethanol to trap several tienilic acid isomer reactive metabolites 10 mM [27]. *N*-Acetyl-cysteine (typically used in vitro at 5 mM) inhibited the major CYP isoforms with the exception of CYP2C9, with IC_50_ values ranging from 9.3–23 mM. *N*-Acetyl-lysine did not show inhibition at concentrations up to 50 mM and a commonly used 1:1 mixture of *N*-acetyl-lysine:*N*-acetyl-cysteine (both agents 50 mM final concentration) inhibited the major CYP isoforms with IC_50_ values comparable to that of *N*-acetyl-cysteine alone, ranging from 8.4–41 mM.

Of the three reducing agents tested (arsenite, dithiothreitol (DTT), and TCEP), both DTT and TCEP showed inhibition against various CYP isoforms. The IC_50_ values of DTT (typically used in vitro at 4 mM) ranged from 3.3–22 mM. The IC_50_ values of TCEP (typically used in vitro at 4 mM) ranged from 1.3–32 mM, with inhibition apparent in all isoforms except CYP2D6 (activation was observed for this isoform). Arsenite (typically used in vitro at 20 mM) did not show inhibition at concentrations up to 10 mM.

Ascorbic acid, a radical scavenger and a reducing agent, inhibited all isoforms except CYP2C9, with IC_50_ values ranging from 2.7–16 mM.

Both NEM and *N*-methylmaleimide, which can be used as Michaël acceptors (for thiol trapping) and dienophiles (for Diels Alder cycloaddition of thiophene *S*-oxides), showed inhibition of all tested isoforms except CYP2D6. The IC_50_ values in order of potency for NEM (typically used in vitro at 4 mM) were 0.12 mM (CYP1A2), 0.14 mM (CYP3A; testosterone), 2.7 mM (CYP3A; midazolam), 2.9 mM (CYP2C19) and 3.2 mM (CYP2C9). Activation was observed for CYP2C9 (at concentrations up to 1 mM) and CYP2C19 (at concentrations up to 0.4 mM). The IC_50_ values in order of potency for *N*-methylmaleimide (typically used in vitro at 4 mM) were 0.20 mM (CYP1A2), 1.5 mM (CYP2C19), 3.3 mM (CYP3A; testosterone), 6.2 mM (CYP2C9) and 12 mM (CYP3A; midazolam). Similar to NEM, *N*-methylmaleimide also showed activation of CYP2C9 and CYP2C19, at concentrations up to 0.2 mM in both cases. It has been reported that CYP3A4 is singly modified at C64 by various maleimide derivatives, which is consistent with our observation of CYP3A inhibition by these two agents [28].

### 2.2. Trapping of Reactive Metabolites of Ticlopidine

The effect of CYP inhibition by a trapping agent (NEM) on CYP-mediated reactive metabolite formation of ticlopidine was investigated. Figure 2b depicts ticlopidine metabolism and this analysis specifically focuses on the formation of TSOD and TSO-NEM. When NEM is absent from the incubation, the reactive intermediate TSO is able to undergo dimerization to form TSOD. With increasing concentrations of NEM (0.007–40 mM) in the incubation, TSOD formation decreases as the reactive TSO intermediate is trapped by NEM and unable to dimerize (Figure 2b). Additionally, with increasing concentrations of NEM used to trap the reactive TSO intermediate, formation of the stable TSO-NEM conjugate initially increases then rapidly decreases at concentrations of 165 μM NEM and above (Figure 2b). The decrease in the amount of trapped reactive metabolite TSO-NEM demonstrates that CYP inhibition by trapping agent NEM can drastically alter the amount of trapped and detected reactive intermediates. This observation was also consistent with reports of TSOD formation catalyzed by CYP2C19 [25], as NEM is an inhibitor of CYP2C19 with an IC_50_ of 2.9 mM (Table 1). The possibility exists that this decrease is due to further reaction of the TSO-NEM adduct with excess NEM; however, no such adduct was detected by LC-MS/MS and no known instances of NEM dimerization exist in the literature, thus this scenario is unlikely.

## 3. Discussion

**Figure 3:**
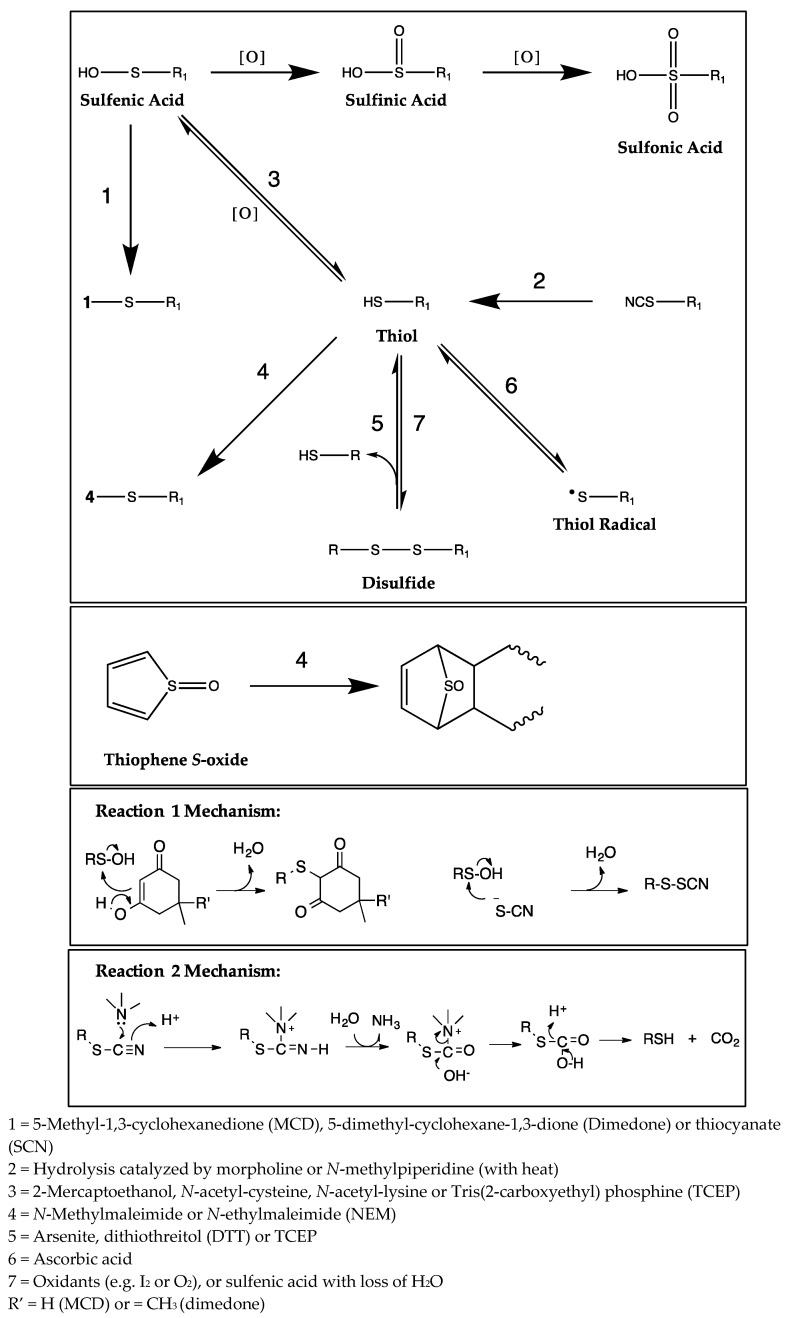
Reactions mediated by trapping agents commonly used to trap sulfur-containing reactive intermediates.

In in vitro experiments, a number of trapping agents are commonly used to assess the formation of reactive intermediates of sulfur-containing drugs. Concentrations of these trapping agents are often high and range from 4–20 mM. Since CYPs are mainly responsible for known bioactivation reactions, it is important to ensure that high concentrations of trapping agents are not inhibiting the activities of CYP isoforms, thereby modulating the CYP-mediated metabolism of the compound of interest and underestimating the formation of potential reactive metabolites. Here we report on the inhibitory potential against the major CYP isoforms (1A2, 2C9, 2C19, 2D6 and 3A) by a broad selection of fourteen trapping agents (including nucleophiles, reducing agents, Michaël acceptors/dienophiles and radical scavengers) used to assess formation of sulfenic acid and its derivatives (Table 1). The chemical structures of the fourteen trapping agents used here are shown in Figure 1 and the common reactions mediated by these agents are shown in Figure 3. Although the perspective of this manuscript focused primarily on reactive metabolites generated from sulfur-containing drugs, it is important to note that the trapping agents tested here can be used to trap reactive intermediates of a wide variety of test compounds.

CYP inhibition of various isoforms was observed for eleven of the fourteen trapping agents at concentrations tested (Table 1). It is noteworthy that in many cases, observed IC_50_ values are lower than concentrations typically used in in vitro trapping experiments. To further investigate the implications of the inhibitory properties of trapping agents on reactive metabolite formation, trapping experiments were performed with NEM, an inhibitor of CYP1A2, CYP2C9, CYP2C19 and CYP3A (Table 1) and ticlopidine. Ticlopidine is known to be metabolized by multiple CYP isoforms, including CYP2C19, CYP2D6 and CYP3A4 [25,26]. There were six metabolites monitored, which have been previously reported [26], and this analysis focused on the effects of NEM on formation of TSOD and TSO-NEM (Figure 2a). In the absence of NEM, the reactive intermediate ticlopidine *S*-oxide was able to undergo dimerization to form TSOD. In the presence of NEM, a stable Diels Alder adduct of ticlopidine *S*-oxide was formed, resulting in TSO-NEM. As expected, the formation of TSOD sharply decreased with increasing concentrations of NEM in the incubation, as the reactive ticlopidine *S*-oxide intermediate was trapped by NEM and unable to dimerize to form TSOD (Figure 2b). With increasing concentrations of NEM in the trapping incubation, TSO-NEM formation initially increased, but rapidly decreased at concentrations of 165 μM NEM and above. This observation demonstrates that addition of higher concentrations of NEM (above 165 μM) into the trapping incubation causes inhibition of ticlopidine metabolism, thereby limiting the formation of the reactive TSO species and results in an overall underestimation of reactive metabolite formation. These results are significant, as NEM is routinely included in trapping incubations at concentrations up to 4 mM. Additionally, it is interesting to note that activation of CYP2C19 by NEM was observed at concentrations up to 0.4 mM (Table 1) and is likely responsible for the initial increase in TSOD formation observed in incubations containing 0.7–18.3 μM NEM (Figure 2b). In the case of ticlopidine, although maximum adduct formation is not achieved at lower concentrations of NEM (less than 165 μM), sufficient assay sensitivity exists to detect TSO-NEM and therefore infer presence of reactive metabolite. This may or may not be the case for all candidate drugs, and therefore should also be taken into consideration when performing trapping studies. The trapping agents tested in this work are essential tools for drug discovery scientists and have key implications for new compounds which contain sulfur atoms (see Figure 3). Commonly formed reactive derivatives of sulfur-containing compounds include sulfenic acid, thiophene *S*-oxide thioamide *S*-oxide, thiocarbamate *S*-oxide and thioxon, and are often produced as a result of CYP-mediated metabolism. Sulfenic acid, for example, is a reactive species which can be formed in three distinct chemical situations depending on the xenobiotic structure: (1) the direct *S*-hydroxylation of a free thiol (RSH), (2) the *S*-oxidation of thioesters (RCOSR′) leading to an oxidative cleavage of their CO-S bond, and (3) the cleavage of a C-S bond of xenobiotics bearing a sulfoxide function, resulting from the attack of a nucleophile [11,29,30,31,32]. Due to the highly reactive characteristics of reactive metabolites such as sulfenic acid, detection is crucial in understanding potential for toxicity of new molecular entities in drug discovery.

A variety of marketed drugs have sulfur-containing moieties, such as thiols, thiophenes, sulfonamides, thioesters, thiocarbamates, thiazolidinediones, thioureas and thiazoles [33,34]. Metabolism of such moieties by CYP isoforms often results in formation of reactive intermediates that may covalently bind to cellular macromolecules such as proteins or DNA, resulting in toxicity (or in some cases pharmacological effect) of parent xenobiotic [11]. For instance, the reactive metabolite sulfenic acid can covalently modify proteins critical in cell function, potentially resulting in toxicity. In contrast, sulfenic acid is not always the culprit of toxicity, but may also be responsible for the desired effect. Clopidogrel, for example, is a prodrug metabolized by CYP2C19 to form a pharmacologically active thiol metabolite that irreversibly inactivates P2Y_12_, a G protein-coupled receptor important in regulating platelet aggregation [35]. This thiol metabolite is formed through three reactions, starting with two successive CYP oxidations (to form a thiolactone then a thiolactone *S*-oxide), which further hydrolyzes into a reactive sulfenic acid and reduces to the active thiol [29,31,32]. Additionally, sulfenic acid formation from cysteine residues in enzymes has been shown to be involved in sensing oxidative stress in enzymes as well as transcriptional regulators [36]. In each of these cases, preclinical detection of reactive metabolite formation is critical in understanding potential toxicological or therapeutic effects of the compound of interest.

The results presented here show that it is imperative to consider concentrations of trapping agents used and CYP isoforms inhibited in in vitro trapping incubations to accurately measure and interpret extent of reactive metabolite formation. Inhibition of CYP enzymes by trapping agents can result in an underestimation of potential reactive metabolite formation and therefore a range of concentrations of trapping agent should be used in trapping studies to ensure optimal trapping of reactive intermediates is achieved. We observed that at concentrations typically used in in vitro trapping studies in HLMs, a majority of the commonly used trapping agents showed inhibition of one or more of the major CYP isoforms. Use of these agents in trapping incubations at typical concentrations used industry-wide may result in reduced amount of reactive metabolite formed and detected. It is noteworthy that in some cases addition of the trapping agent resulted in higher CYP metabolic activity than the vehicle control incubation, indicating activation of that CYP isoform (denoted in Table 1 with an underlined IC_50_ value). The activation of a CYP isoform responsible for generating a reactive metabolite will result in an increased amount of reactive species formed, trapped and detected, resulting in an overestimation of actual formation. Additionally, consideration of which CYP isoforms are involved in metabolism of drug-of-interest will further assist in selection of trapping agent. The results presented in this manuscript will act as a guide for specific agents to use for trapping reactive sulfur-containing metabolites, as well as defining concentrations at which agents should be used to minimize CYP inhibition (or activation).

## 4. Materials and Methods

### 4.1. Materials

Pooled human liver microsomes (HLM; both male and female; 150 donors; protein concentration of 20 mg/mL) were purchased from BD Biosciences (San Jose, CA, USA) and stored at −80 ^o^C. Potassium phosphate buffer (KPi; pH 7.4; 1.0 M) was provided by the Media Preparation Facility at Genentech, Inc. (South San Francisco, CA, USA). The incubation buffer was prepared by diluting 1 M KPi buffer solution in water to 100 mM KPi. l-Ascorbic acid, dextromethorphan, dimethylsulfoxide (DMSO), formic acid, *N*-methylpiperidine, 5-methyl-1,3-cyclohexanedione (MCD), morpholine, NADPH, testosterone, thiocyanate (SCN), ticlopidine and (*S*)-warfarin were purchased from Sigma-Aldrich (St. Louis, MO, USA). Tacrine was purchased from Fluka Chemika (Buchs, Switzerland). Dextrorphan-D_3_ and 6β-hydroxytestosterone-D_3_ were purchased from Cerilliant (Round Rock, TX, USA). Midazolam was purchased from Spectrum Chemicals (Gardena, CA, USA). 1′-Hydroxymidazolam-D_3_ was purchased from High Standard Products (Westminster, CA, USA). 4′-Hydroxymephenytoin-D_3_ and phenyl-D_5_-7-hydroxywarfarin were purchased from BD Biosciences. Hydroxytacrine-D_4_ and (*S*)-mephenytoin were purchased from Toronto Research Chemicals (North York, Ontario, Canada). Acetonitrile (ACN) was purchased from Honeywell Burdick & Jackson (Muskegon, MI, USA). *N*-Acetyl-l-cysteine and dithiothreitol (DTT) were purchased from Axxora, LLC (San Diego, CA, USA). Tris(2-carboxyethyl)phosphine (TCEP) was purchased from EMD Biosciences, Inc. (San Diego, CA, USA). *N*-Acetyl-lysine, sodium arsenite, 5,5-dimethyl-1,3-cyclohexanedione (dimedone), *N*-ethylmaleimide (NEM) and *N*-methylmaleimide were purchased from MP Biomedicals (Solon, OH, USA). 2-Mercaptoethanol was purchased from TCI America (Portland, OR, USA). An internal standard (IS) from an in-house proprietary compound was provided by Genentech, Inc.

Matrix 96w 0.5 mL 2D Barcoded tubes and V Bottom ScrewTop tubes were purchased from Matrix Technologies (Hudson, NH, USA). Greiner 384w polypropylene microplate, Greiner 384 deep well V-bottom plates, and ThermoScientific Universal microplate lids were purchased from VWR International (West Chester, PA, USA)). Reservoirs (300 mL) were purchased from E&K Scientific Products (Santa Clara, CA, USA)). Agilent 96LT 200 µL tip boxes and Agilent 384ST 70 µL tip boxes were purchased from Agilent Technologies, Inc. (Wilmington, DE, USA)).

### 4.2. CYP Inhibition

The automated 384-well CYP inhibition experiment was performed as described previously, amended to accommodate manual compound dilution to maximize highest-tested concentration of each agent [37]. In brief, each trapping agent was incubated at various test concentrations in HLM fortified with NADPH in six separate reactions containing CYP-probe substrates, each selective for a specific CYP isoform (1A2, 2C9, 2C19, 2D6 or 3A). CYP inhibition incubation conditions are based on validated enzyme kinetic conditions specific for each probe substrate that are consistent with regulatory guidance (Table 2). The concentration range of each agent was selected to include concentrations commonly used in in vitro trapping experiments (see Table 1) and when aqueous solubility permitted, agents were tested up to 100 mM. In addition, individual incubations were visually inspected throughout the experiment to ensure trapping agent had not fallen out of solution. In these cases, the experiment was repeated at a lower concentration range. Arsenite was tested at concentrations lower than typically used in vitro due to limitations in aqueous solubility. Each agent was assayed at three different starting concentrations in the selected concentration range, in triplicate, with each starting concentration diluted 2-, 4-, 8-, 16-, 32- and 64-fold, as well as an incubation containing no compound (vehicle control), to generate the half maximal inhibitory concentration (IC_50_) values.

### 4.3. Trapping of Reactive Metabolites of Ticlopidine

Ticlopidine (1 μM) was incubated at 37 °C for 15 min in the presence or absence of various concentrations of NEM with HLM (0.5 mg/mL), NADPH (1 mM) and KPi (0.1 M), in a final incubation volume of 100 µL. Assay conditions were optimized with regard to protein concentration and incubation time, based on linear formation of TSOD. The incubations were performed over a concentration range of NEM (0.0007–40 mM) including a vehicle control, for a total of twelve incubations. A 50 µL sample was withdrawn at 15 min and the reactions were quenched in 100 µL of 0.1% formic acid in ACN containing an IS. Samples were centrifuged at 2000× *g* for 10 min and supernatants (75 µL) were removed and diluted with 0.1% formic acid in water (150 µL). The prepared samples were analyzed by LC-MS/MS for ticlopidine, *N*-ethylmaleimide-trapped *S*-oxide (TSO-NEM) and ticlopidine *S*-oxide dimer (TSOD). Peak areas were normalized to an IS, and the percentage of metabolite formed at the end of the incubation was calculated and compared to the incubation containing the highest peak area to IS ratio of metabolite (set at 100%).

### 4.4. LC-MS/MS Analysis

The prepared samples were analyzed by LC-MS/MS using a Cohesive LX-2 Transcend Multiplexing system with Agilent 1100 series high pressure liquid chromatography (HPLC) pumps from Agilent Technologies (Santa Clara, CA, USA) and an HTS PAL autosampler from CTC Analytics (Carrboro, NC, USA) connected to a Sciex API5500-QTrap mass spectrometer with a Turbo V™ ion source (Foster City, CA, USA). A Phenomenex Kinetex Phenyl-Hexyl HPLC column (30 × 2.1 mm, 2.6 µ) was used with 0.1% formic acid in water (mobile phase A) or 0.1% formic acid in ACN (mobile phase B) flowing at 0.55 mL/min in the following gradient: mobile phase B was held at 1% for 0.4 min, increased to 5% over 0.2 min, to 55% over 6.7 min and to 95% over 0.5 min, where it was held for 0.75 min prior to re-equilibration at 1%. LC-MS/MS multiple reaction (MRM) transitions for the CYP inhibition assay were reported previously [37]. The MRM transitions for the ticlopidine trapping samples were *m*/*z* 264→154 (ticlopidine), *m*/*z* 405→357 (TSO-NEM) and *m*/*z* 559→511 (TSOD).

### 4.5. Data Analysis

IC_50_ data analyses were performed using the GraphPad Prism 5.0 by Graphpad Software, Inc. (La Jolla, CA, USA). The log inhibitor concentration versus normalized response calculation was used to determine IC_50_ values for each trapping agent against each major CYP isoform, with two significant figures. Each trapping agent was assessed in triplicate and average IC_50_ values plus 95% confidence intervals were calculated and reported in Table 1. Potential activation, where metabolite formation increased with increasing concentrations of agent, was noted as an underlined IC_50_ value.

## Figures and Tables

**Table 1:** Inhibition of trapping agents against the major cytochrome P450 (CYP) isoforms.

Class	Trapping Agent	Typically Used In Vitro Concentration ^1^ (mM)	Highest Tested Concentration (mM)	IC_50_ (mM) (95% Confidence Interval)					
				CYP1A2	CYP2C9	CYP2C19	CYP2D6	CYP3A_T ^2^	CYP3A_M ^3^
Nucleophiles	Morpholine	10	100	>100	7.9 (6.0–10.1)	14 (11–16)	13 (11–16)	16 (15–18)	9.1 (7.3–11.5)
	*N*-Methylpiperidine	10	100	~50 ^4,a^	9.9 (8.0–12.1)	19 (13–28)	6.5 (5.7–7.4)	14 (12–16)	12 (11–14)
	5-Methyl-1,3-cyclohexanedione (MCD)	4	10	3.6 (2.9–4.3)	6.7 (5.4–8.3)	3.1 (2.1–4.7)	>10	5.5 (3.7–8.1)	>10
	5-Dimethyl-cyclohexane 1,3-dione (Dimedone)	1–4	5	>5	>5	>5	>5	>5	>5
	Thiocyanate (SCN)	4	100	>100	18 (14–22)	20 (13–31)	49 (42–59)	43 (34–54)	46 (35–61)
	2-Mercaptoethanol	20	100	42 (26–67)	2.0 (1.8–2.3)	0.33 (0.30–0.36)	24 (22–27)	7.8 (6.5–9.4)	8.9 (6.3–12.7)
	*N*-Acetyl-cysteine1	5	50	9.3 (7.8–11.0)	>50	18 (15–22)	23 (20–25)	15 (13–18)	20 (15–26)
	*N*-Acetyl-lysine1	5	50	>50	>50	>50	>50	>50	>50
	*N*-Acetyl-lysine: *N*-Acetyl-cysteine	5:5	50:50	4.3 (3.7–5.1)	41 (18–96)	14 (12–16)	14 (11–17)	13 (11–15)	8.4 (7.0–10.1)
Reducing Agents	Arsenite	20	10	>10	>10	>10	>10	>10	>10
	Tris(2-carboxyethyl) phosphine (TCEP)	4	100	3.4 (1.2–9.9)	32 (13–79)	1.3 (0.1–17.1)	>100	8.8 (8.0–9.7)	20 (17–24)
	Dithiothreitol (DTT)	4	100	16 (13–19)	7.2 (6.2–8.3)	5.6 (5.0–6.3)	22 (19–25)	3.3 (2.7–4.0)	9.4 (8.9–9.9)
Michaël Acceptors and Dienophiles	*N*-Ethylmaleimide (NEM)	4	40	0.12 (0.01–0.14)	3.2 ^4,b^(0.7–14.7)	2.9 ^4,c^(1.2–7.1)	>40	0.14 (0.13–0.16)	2.7 (2.4–3.1)
	*N*-Methylmaleimide	4	20	0.20 (0.16–0.26)	6.2 ^4,d^(0.8–51.0)	1.5 ^4,d^(0.6–3.5)	>20	3.3 (2.6–4.4)	12 (9–15)
Radical Scavenger	Ascorbic Acid	3	50	16 (13–20)	>50	16 (7–37)	12 (8–18)	2.7 (1.6–4.9)	11 (8–16)

Underlined IC_50_ values indicate activation of that CYP isoform was observed; ^1^ Concentration of trapping agent commonly used in in vitro trapping studies; ^2^ CYP3A_T = CYP3A and testosterone as the probe substrate; ^3^ CYP3A_M = CYP3A and midazolam as the probe substrate; ^4^ Activation observed at concentrations up to ^a^ 12.5 mM, ^b^ 1 mM, ^c^ 0.4 mM, ^d^ 0.2 mM.

**Table 2:** Final reaction conditions for CYP inhibition assays.

CYP Isoform	Probe Substrate (Concentration in µM)	Metabolite Monitored	Incubation Time (min)	Human Liver Microsomal Concentration (mg/mL)
1A2	Tacrine (1)	1′-Hydroxytacrine	10	0.2
2C9	(*S*)-Warfarin (2)	7-Hydroxywarfarin	30	0.2
2C19	(*S*)-Mephenytoin (60)	4′-Hydroxymephenytoin	40	0.2
2D6	Dextromethorphan (5)	Dextrorphan	10	0.03
3A	Midazolam (2)	1′-Hydroxymidazolam	10	0.03
3A	Testosterone (50)	6β-Hydroxytestosterone	10	0.07

## Abbreviations

CYP	Cytochrome P450
Dimedone	5,5-Dimethyl-1,3-cyclohexanedione
DTT	Dithiothreitol
GSH	Glutathione
HLM	Human liver microsomes
LC-MS/MS	Liquid chromatography-tandem mass spectrometry
MCD	Methyl-cyclohexane 1,3-dione
NADPH	Reduced β-nicotinamide adenine dinucleotide phosphate
NEM	*N*-Ethylmaleimide
SCN	Thiocyanate
TCEP	Tris(2-carboxyethyl) phosphine
TSO	Ticlopidine *S*-oxide
TSOD	Ticlopidine *S*-oxide dimer
TSO-NEM	*N*-ethylmaleimide ticlopidine *S*-oxide adduct
TCEP	Tris(2-carboxyethyl) phosphine
